# Hemodynamic responses on prefrontal cortex related to meditation and attentional task

**DOI:** 10.3389/fnsys.2014.00252

**Published:** 2015-02-17

**Authors:** Singh Deepeshwar, Suhas Ashok Vinchurkar, Naveen Kalkuni Visweswaraiah, Hongasandra RamaRao Nagendra

**Affiliations:** ANVESANA Research Laboratory, Department of Yoga and Life Sciences, Swami Vivekananda Yoga Research FoundationBangalore, Karnataka, India

**Keywords:** meditation, attention task, Stroop task, fNIRS, cerebral blood flow

## Abstract

Recent neuroimaging studies state that meditation increases regional cerebral blood flow (rCBF) in the prefrontal cortex (PFC). The present study employed functional near infrared spectroscopy (fNIRS) to evaluate the relative hemodynamic changes in PFC during a cognitive task. Twenty-two healthy male volunteers with ages between 18 and 30 years (group mean age ± SD; 22.9 ± 4.6 years) performed a color-word stroop task before and after 20 min of meditation and random thinking. Repeated measures ANOVA was performed followed by a *post hoc* analysis with Bonferroni adjustment for multiple comparisons between the mean values of “During” and “Post” with “Pre” state. During meditation there was an increased in oxy-hemoglobin (ΔHbO) and total hemoglobin (ΔTHC) concentration with reduced deoxy-hemoglobin (ΔHbR) concentration over the right prefrontal cortex (rPFC), whereas in random thinking there was increased ΔHbR with reduced total hemoglobin concentration on the rPFC. The mean reaction time (RT) was shorter during stroop color word task with concomitant reduction in ΔTHC after meditation, suggestive of improved performance and efficiency in task related to attention. Our findings demonstrated that meditation increased cerebral oxygenation and enhanced performance, which was associated with activation of the PFC.

## Introduction

Meditation is a complex mental process that aims to calm the fluctuations of the mind and improve cognitive functions. Several meditation techniques from diverse traditions (e.g., Transcendental meditation, Buddhists, Zen, Yoga, Vipassana, Brahmakumari, Mindfulness-based stress reduction (MBSR) etc.,) demonstrated that regular practice of meditation develops awareness to the contents of subjective experience, including thoughts, sensations, intentions, and emotions (Saggar et al., 2012). It is considered as a voluntary means of mental training to achieve greater control of higher mental functions. Traditional yoga texts like Patanjali’s Yoga *Sutras* (the Sage Patanjali’, *Circa* 900 B.C.) and *Bhagavad Gita* (*Circa* 400–600 B.C.) very well describe the connection between meditation and mental modifications. Traditionally, two states of meditation have been described, viz., (i) focused meditation (*dharana* in Sanskrit, Patanjali’s Yoga *Sutras*, Chapter III, Verse 1), and this state is supposed to lead to the next stage of effortless mental expansion i.e., (iii) meditation (*dhyana* in Sanskrit; Patanjali’s Yoga *Sutras*, Chapter III, Verse 2). When not in meditation, it is said that the mind may be in two other states (Telles et al., 2012). These are (i) random thinking (*cancalata* in Sanskrit; *Bhagavad Gita*, chapter VI, *verse* 34); and (ii) non-meditative focused thinking (*ekagrata* in Sanskrit; *Bhagavad Gita*, chapter VI, *verse* 12) (Telles et al., 2014).

In recent years, there have been a number of neuroimaging studies showing that meditation improves cognitive performance as signified by behavioral and neurophysiological measures (Tang et al., 2007; Lutz et al., 2009). Previous studies have shown that the practice of meditation enhances behavioral performance viz., perceptual discrimination and sustained attention during visual discrimination task (MacLean et al., 2010). Meditation practice develops the ability to engage the attention onto an object for extended periods of time (Carter et al., 2005; Jha et al., 2007; Lutz et al., 2008). It improves the control over the distribution of limited brain resources in the temporal domain, as measured by the attentional blink task (van Leeuwen et al., 2009; Slagter et al., 2011). Long term meditation practice has been found to enhance cognitive performance (Cahn and Polich, 2006), attentional focus, alerting (Jha et al., 2007), processing speed (Lutz et al., 2009; Slagter et al., 2009), and overall information processing (van Vugt and Jha, 2011). In a study, Buddhist meditation practitioners showed mindfulness meditation was positively correlated with sustained attention, when compared to non-meditation practitioners (Moore and Malinowski, 2009). Improvements in sustained attention and attentional error monitoring demonstrated a positive correlation with increased activation in executive attention networks in meditators (Short et al., 2010). Other studies have shown that meditation is associated with improved conflict scores on the attention network test (Tang et al., 2007), reduced interference (Chan and Woollacott, 2007) and enhanced attentional performance during the stroop task compared to meditation-naïve control group (Moore and Malinowski, 2009). These studies provide significant evidence of meditation promoting the higher-order cognitive processing (Zeidan et al., 2010), particularly, the features of conflict monitoring and cognitive control processes.

The stroop task is one of the most frequently used models of the conflict processing (Szűcs et al., 2012) in cognitive neuroscience. Stroop color word task performance evaluates flexibility in the purview of cognitive processes and behavior which requires both attention and impulse control. The simultaneous presentation of the prime color and a written word stimulus will either facilitate (when the color and word stimuli are congruent, e.g., “b-l-u-e” written in the color blue) or interfere (the incongruent stroop trial, e.g., “blue” written in red) with color naming (MacLeod, 1991; Peterson et al., 1999). Previous studies on stroop test have consistently shown that responses in naming the ink color of incongruent color word are much slower than in naming the ink color of neutral (Zysset et al., 2007), and responses are often, but not always, faster when color and word are congruent than in the neutral condition. It supports the hypothesis that, both the task relevant and task irrelevant dimensions of stroop task activate the same response in the congruent condition, in contrast, these dimensions stimulate opposing response tendencies in the incongruent condition (Morton and Chambers, 1973; Posner and Snyder, 1975; Szűcs et al., 2012).

Recent studies reported that regular practice of meditation may alter brain structure and function related to attention (Lazar et al., 2005; Holzel et al., 2011; Kozasa et al., 2012). A study on 20 experienced participants of extensive Insight meditation, that involves focused attention to internal experiences, reported increased cortical thickness in prefrontal cortex (PFC) and right anterior insula associated with attention, interoception and sensory processing in meditation participants compared with matched controls (Lazar et al., 2005).

In order to examine neuronal activity and hemodynamic changes in the brain regions during meditation, the application of different neuroimaging techniques (viz., fMRI and MEG) would be beneficial. The neuronal activity during meditation has been reported in several electroencephalography (EEG) and magnetoencephalography (MEG) studies. Experienced meditators showed an increased EEG power in lower frequency bands (theta, delta and alpha) (Kubota et al., 2001; Takahashi et al., 2005) compared to controls. An EEG study on Transcendental Meditation, showed intermittent prominent bursts of frontally dominant theta activity at an average maximal amplitude of 135 µV in 21 practitioners (Hebert and Lehmann, 1977). Zen meditators showed fast theta and slow alpha power during meditation (Takahashi et al., 2005) demonstrating enhanced automatic memory and reduction in conceptual thinking following meditation (Faber et al., 2014). In a single MEG study on twelve long term Buddhist meditators were assessed in two distinct types of self-awareness, i.e., “narrative” and “minimal” in mindfulness-induced selflessness awareness (Dor-Ziderman et al., 2013). It was found that there was a reduction in gamma band (60–80 Hz) power in frontal, and medial prefrontal areas, and reduced beta band (13–25 Hz) power in ventral medial prefrontal, medial posterior and lateral parietal regions (Dor-Ziderman et al., 2013) and right inferior parietal lobules. These studies are consistent with fMRI and NIRS findings. Functional magnetic resonance imaging (fMRI) poses several challenges such as high sensitivity to participant’s motion, a loud, restrictive environment, low temporal resolution, and relatively high cost (Cui et al., 2011). Some of these challenges are overcome with new optical imaging technique: NIRS measure’s changes in oxy-hemoglobin and deoxy-hemoglobin (ΔHbO and ΔHbR) concentration changes from the cortical surface and less invasive and expensive than fMRI (Bunce et al., 2006). Functional near infrared spectroscopy (fNIRS) is a compact and portable optical technique to monitor hemodynamics of the brain in real time (Son and Yazici, 2006; Lin et al., 2009).

Brain hemodynamic responses during meditation, i.e., ΔHbO, ΔHbR and total hemoglobin changes (ΔTHC) are in its infancy. In fact, there is only one study that assessed deoxyhemoglobin changes with a single wavelength probe placed over the left PFC during Qigong meditation (Cheng et al., 2010). Practitioners showed decrease in deoxy-hemoglobin and increase in oxy-hemoglobin concentration that suggest, meditation lead to left prefrontal activation during meditation.

With this background, the present study was designed to assess the bilateral prefrontal hemodynamic responses in meditation and random thinking. Additionally, we investigated the hemodynamic changes and performance during a stroop color word task before and after meditation and random thinking. Since, stroop color word task is known to measure attention, interference, processing speed, and executive attention, we expected that this task to be the most sensitive to the effects of meditation.

## Materials and methods

### Participants

A total of 25 right handed healthy male participants with ages ranging from 19 and 30 years (Mean, SD; 23.4 ± 3.7 years) were recruited from S-VYASA (a Yoga University), South India. All participants had a minimum of 12-month experience in meditation (group average experience ± S.D., 15.6 ± 14.2 months) on the Sanskrit syllable “*OM*”. Three participants were excluded from the study because of large motion artifacts in the signals due to head movements or because of failure in probe placement due to obstruction by hair (Taga et al., 2003; Minagawa-Kawai et al., 2011). Thus, only data from 22 participants (mean age 22.9 ± 4.6 years) were included in the final analysis. Participants fulfilling the following criteria were included in the study: (i) the participants with at least 12 months of meditation experience; (ii) male participants alone were studied as cognitive abilities and cerebral blood flow (Brackley et al., 1999) have been shown to fluctuate which the phases of menstrual cycle (Yadav et al., 2002); and (iii) no history of smoking; and (iv) normal health on a routine clinical examination. Participants with following criteria were excluded from the study: (i) persons on any medication or herbal remedy; (ii) participants having clinical evidence of medical, neuropsychological, or drug abuse that would potentially alter cerebral blood flow (Liddle et al., 1992; Newberg et al., 2010a,b; Goldstein and Volkow, 2011); and (iii) any visual deficit; and (iv) any cognitive impairment. None of the potential participants were involved in any other ongoing research activity. The characteristics of participants are given in Table 1.

**Table 1:** **Characteristics of 22 participants**.

Characteristics
**Age (in years) (group mean ± S.D.)**	22.9 ± 4.6 years
**Years of education**	
17 years and more	6 (27.3%)
Upto 15 years	10 (45.5%)
Upto 12 years	6 (27.3%)
**Type of meditation**	Meditation on the Sanskrit syllable “*OM*”
**Experience of meditation practice (in months)**	
6–12 months	4 (18.2%)
13–24 months	3 (13.6%)
25–36 months	7 (31.8%)
37–48 months	6 (27.3%)
48–60 months	2 (9.1%)

The study was approved by the Institutional Ethics Committee of S-VYASA, a Yoga University (No.-RES/IEC-S-VYASA/11/2011). The study protocol, nature of the experiments and the operating mode of the instrument was explained to the subjects before obtaining signed informed consent.

### Design

**Figure 1:**
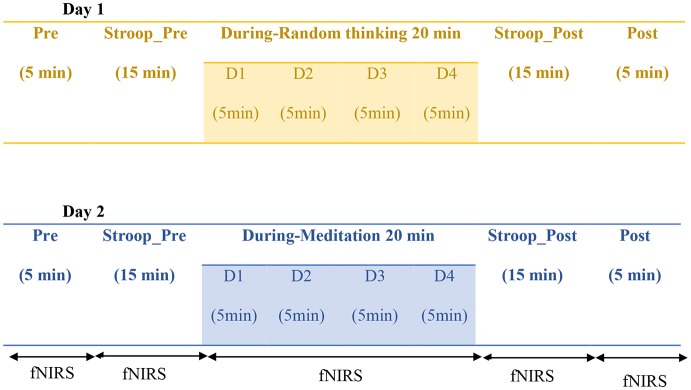
**Schematic representation of the study design. Note**: Sessions were modified for each participant D1: During 1; D2: During 2; D3: During 3; D4: During 4.

The protocol utilized in the present study consisted of two sessions i.e., random thinking (*cancalata*) and meditation (*dhyana*), and eight States (Pre, Stroop_Pre, During (D1-D4 each of 5 min), Stroop_Post, and Post). Each participant was assessed for both the meditation and control session on two separate consecutive days. The sessions were randomized online with randomization software.^1^ During the acquisition and analysis of data, researcher was blinded to the session of the individual. The total duration of the each session was 60 min: Pre (5 min), Stroop_Pre (15 min), During (20 min), Stroop_Post (15 min), and Post (5 min). The schematic presentation of the design has been given in Figure 1.

Apart from their prior experience of meditation on “*OM*”, all participants were given a 3 month orientation, 5 days a week under the guidance of an experienced meditation teacher. The purpose of this orientation was for to ensure uniformity among all practitioners based on specific instructions.

### Interventions

Each participant sat cross-legged with eyes closed and followed pre-recorded instructions throughout meditation and random thinking sessions. An emphasis was placed on slowly, practice with awareness of physical and mental sensations, and relaxation. The duration of each session was 20 min between 06:00 to 06:30 h conducted 5 days a week. The theoretical aspects of the meditation were detailed by the meditation teacher on the first day. Following this, the practice of each session began with pre-recorded instructions. The practice of meditation was evaluated based on their self-reporting and by consultations with the meditation teacher. The two phases—random thinking (Rand) and meditative defocusing were as follows:

1. ***Random thinking***:
  Participants were asked to listen a compiled audio CD consisting of brief periods of random conversation, announcements, various advertisements and non-connected talks recorded from a local radio station transmission and allow their thoughts to wander freely. All these non-connected conversations could induce the state of random thinking.
2. ***Meditative de-focusing or effortless meditation***:
  In effortless meditation session, each participant was instructed to dwell effortlessly on thoughts of “*OM*”, particularly on the subtle (rather than physical) attributes and connotations of the syllable with closed eyes. This involved combined mental chanting with effortless defocusing on syllable “*OM*”. This gradually allowed the participants to experience brief periods of silence, which they reported after the session.

### Assessments procedure

#### Laboratory environment

All Participants were assessed in a sound and light dampening Faraday cage. Participants’ were monitored using a closed circuit television outside the cabin to detect if they moved or fell asleep during a session. During the session, instructions were passed through a two-way intercom, so that participants could remain uninterrupted. The recording room temperature was maintained at 24.0 ± 1.0°C with 56 percent average humidity during the conduct of experiments. The background noise level was 26 dB of the acoustically shielded chamber. For each participant, the data acquisition session lasted 60 min.

#### Functional near infrared Spectroscopy (fNIRS)

**Figure 2:**
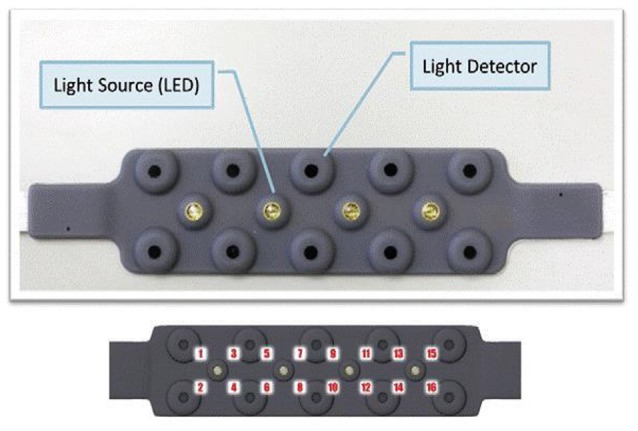
**The complete setup employed is herein presented**. The fNIRS sensor is displayed with 4 light sources and 10 detectors (top) and 16 optode (channel) measurement locations registered on the sensor.

**Figure 3:**
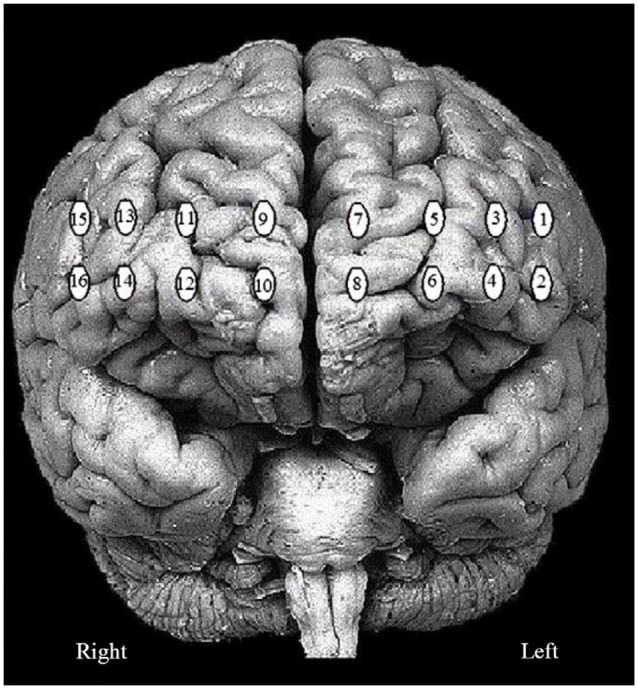
**The 16 fNIRS optode (channel) measurement locations registered on the brain surface image are presented**.

A 16-channel continuous wave fNIRS imager system (FNIR1000-ACK-W, BIOPAC Systems, Inc., U.S.A) was employed to map changes in ΔHbO, ΔHbR and ΔTHC over bilateral PFC. The system consisted of a flexible probe to match contour of the human forehead (see Figure 2). The probe embedded with four LED diodes as light sources (at *λ*_1_ = 730 nm, *λ*_2_ = 830 nm, *λ*_3_ = 850 nm) and ten photodiodes as detectors that were symmetrically arranged in an area of 3.5 × 14 cm^2^, conducing to 16 nearest source—detector (i.e., channels) at 2.5 cm separation displayed in Figure 3. A source-detector distance provides a penetration depth of 1.25 cm (León-Carrion et al., 2008; Kim et al., 2010; Leon-Dominguez et al., 2014). The description of the probe setting is detailed in earlier studies (Krawczyk, 2002; Izzetoglu et al., 2005; Leon-Dominguez et al., 2014). During the experiment, the probe was firmly held with a velcro band on the forehead, and stretched from hairline to eyebrow in a sagittal direction and from ear to ear in axial direction (Tian et al., 2009). The probes were positioned bilaterally on forehead, over the left and right frontal poles, a part of dorsolateral PFC, and a portion of the ventrolateral PFC. Regional cerebral blood flow (rCBF), ΔHbO, ΔHbR, and ΔTHC for each hemisphere were updated every 0.5 s. The four LEDs flashed in sequence; the reflected light from the brain as detected with the nearest photodiodes of each LED and converted into digital signals using an analog-digital converter (ADC) card in the control box. The digital data were sent to the laptop though a serial port. The sampling rate was 3 Hz across all 16 channels. The principles of measurement were based on the modified Beer-Lambert law for highly scattering media (Plichta et al., 2006) that agrees assessing changes in ΔHbO and ΔHbR at a certain measured point (Hoshi and Tamura, 1993). Increases in ΔHbO and corresponding decrease in ΔHbR can be interpreted as a sign of functional brain activation.

#### Stroop color word task

**Figure 4:**
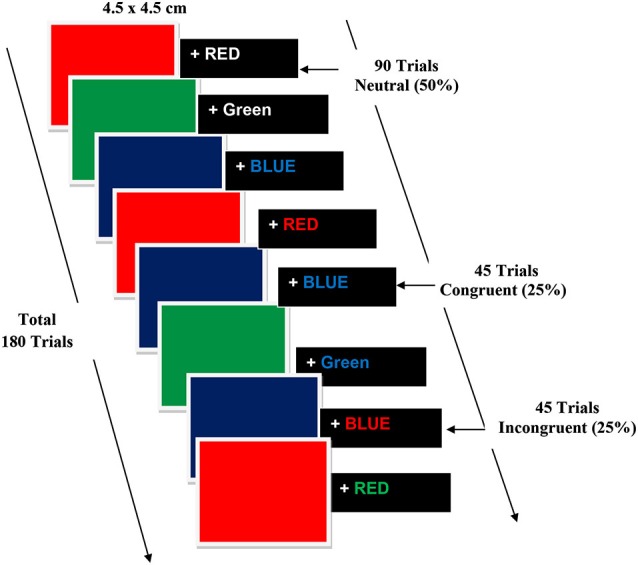
**Experimental steps of Color word Stroop Task**.

Subjects were seated comfortably on a reclining chair in a Faraday cage, facing a 21 inch LCD monitor placed at a distance of 70 cm from their eyes. Participants were required to focus on the center of the screen which was guided by a fixation object “+” followed by stimuli. Participants did a modified multiple-trial stroop task and were confronted with neutral, congruent, and incongruent stimuli on a black background using E-Prime 2.0.8.90 (Psychological Software Tools, Inc., Pittsburgh, PA, USA). The stroop color word task consisted of red, green and blue colored boxes and the corresponding written words “RED”, “BLUE” and “GREEN”. The color was presented as color square (4.5 × 4.5 cm) boxes on a black background. The duration of the presented square boxes and words was 500 ms each. Congruent trials comprised of square color boxes followed by words describing the color of the box written in the same color (e.g., the BLUE square box and the printed word “BLUE” in blue ink); incongruent trials comprised of words describing the color of the box written in a color other than that of the box (e.g., the RED square box and word RED written in blue ink); neutral trials comprised words written in white (e.g., the BLUE square box and word BLUE printed in white ink). Participants were instructed to reply as speedily and accurately as possible to the name of the color word (while ignoring the color itself) consistent to the color of the Box with a button press of the response key using the thumb of their right hand. To increase the potency of the conflict stimulus, 20% of trials were congruent (approximately 45 trials), 20% were incongruent (approximately 45 trials) and 50% were neutral (90 trials). The duration of the stimulus was 500 ms, with a variable interstimulus interval (ISI) of 1000–2500 ms the experimental steps are illustrated in Figure 4.

#### Data acquisition

The participants were assessed in two separate sessions i.e., random thinking and meditation while recording hemodynamic activity on the PFC using 16-channel continuous wave fNIRS system. On the preceding day and on the day of the recording, participants were asked to avoid tea and coffee which are known to influence cognitive performance (Nehlig, 2010) and cerebral blood flow (Addicott et al., 2009). Where this was unavoidable the session was engaged on another day. The participants wore a flexible sensor pad over prefrontal region and covered with a black cloth. The probable artifacts such as heart rate pulsation, respiration and high frequency noise in raw data, which may possibly be induced by autonomic arousal caused during stroop task, was eliminated with pre designed finite impulse response (FIR) filters based on type, order, window function and cut-off frequency. For the present study, raw data were acquired from the probe, which is pre-filtered by two filters and processed in the data processing unit using COBI filter module. The first filter is a 10th order low-pass filter with cutoff frequency of 0.1 Hz with Blackman window. The second filter is a 20th order low-pass, with the normalized cut-off frequency of 0.1 Hz which uses a Hamming window. The filtered data were averaged according to the tasks and conditions for further statistical analysis.

#### Data analysis

The hemodynamic responses of bilateral PFC were recorded and data were averaged according to the task condition (pre, stroop_pre, during, stroop_post and post). Statistical analysis has been carried out on these differential values. Filtered data were tested with Kolmogorov-Smirnov test for normality. Repeated measures analysis of variance (RM-ANOVA) was used because the same individuals were assessed in repeated sessions on two separate days (i.e., random thinking and meditation). RM-ANOVA was performed with three “within subjects” factors, i.e., Factor 1: Sessions (random thinking and meditation); Factor 2: PFC (right and left). Factor 3: States (“Pre”, “Stroop_Pre”, “During” (D1 to D4), “Stroop_Post” and “Post”). The repeated measures ANOVAs were carried out for concentration changes of oxygenated and deoxygenated hemoglobin and total hemoglobin change (ΔHbO, ΔHbR and ΔTHbC) across the right and left PFC. This was followed by a *post hoc* analysis with Bonferroni adjustment for multiple comparisons between the mean values of different states (“During” and “Post”) and all comparisons were made with the respective “Pre” state.

Moreover, for analysis of stroop task we compared the mean reaction time (ms) of neutral, congruent and incongruent conditions and hemodynamic responses of stroop color word task before and after the sessions (random thinking and meditation). The results were averaged for each side of PFC (right and left), parameter and subject separately to compare between different conditions and sessions. A repeated measures ANOVA was carried for multiple comparisons following Bonferroni adjustment. Statistical analyses were carried out using the Statistical software SPSS version 20.0 (SPSS Inc., Chicago, USA). The alpha level was set at *p* < 0.05. The effect size (*d*) defined by Cohen (1988), as the mean change score divided by the standard deviation of change, calculated for further statistical analysis.

## Results

### Behavioral results

Reaction times (RTs) were computed solely from the correctly answered trials. With respect to RT, a repeated—measures 3 way ANOVA with Sessions (random thinking and meditation) × States (“Stroop_Pre”, “Stroop_Post”) × Conditions (neutral vs. congruent vs. incongruent). Repeated measures ANOVA demonstrated a significant main effect for Sessions (*F*_(1,21)_ = 4.862, *p* = 0.039, *η*^2^*p* = 0.188); Conditions (*F*_(2,42)_ = 24.12, *p* < 0.001, *η*^2^*p* = 0.535); States (*F*_(1,21)_ = 6.696, *p* < 0.023, *η*^2^*p* = 0.242), and the significant interaction between Sessions × States (*F*_(1,21)_ = 45.36, *p* < 0.001, *η*^2^*p* = 0.684).

*Post hoc* analysis revealed that there was a significant improvement in cognitive performance after meditation in all three conditions (neutral, congruent and incongruent) compared to random thinking session given in Table 1. The RTs differed in all the conditions (neutral vs. congruent vs. incongruent) in both the sessions. These findings verify that our attentional manipulation was indeed effective.

The RTs were compared using two-tailed paired sample *t*-test, revealed significant differences among all three conditions (neutral, congruent and incongruent) in two different sessions (meditation and random thinking). In random thinking session, there were significant differences in neutral vs. congruent: *t*_(21)_ = −3.86, *p* = 0.001; congruent vs. incongruent: *t*_(21)_ = −2.31, *p* = 0.031; neutral vs. incongruent: *t*_(21)_= −5.92, *p* < 0.001 whereas in meditation session, there was a significant difference in neutral—congruent: *t*_(21)_ = −4.47, *p* < 0.001; congruent—incongruent: *t*_(21)_ = −1.85, *p* > 0.05 (NS); neutral—incongruent: *t*_(21)_ = −6.148, *p* < 0.001. The mean RTs were significantly shorter in the neutral (*p* = 0.002), congruent (*p* < 001) and incongruent (*p* < 0.003) conditions after meditation session whereas after the random thinking session, mean RTs were delayed in the neutral (*p* = 0.034) and incongruent (*p* = 0.008) conditions. The average RTs for neutral, congruent, and incongruent trials of the stroop color word task are given in Table 2. Subjects made negligible errors during the color word matching stroop task. For error rates, we did not make any statistical test, since their distributions are clearly not Gaussian. However, it can be supposed that interference effect also reveals itself in error rates. In summary, behavioral results of the stroop color word task are in accordance with the literature, as demonstrated by a clear interference effect in the participants for meditation and random thinking sessions.

**Table 2:** **Group mean values ± S.D. of the reaction time scores (ms) of Stroop color word Task**.

Sessions	States	Pre	Post	*t*-value	*P* value	% Change
**Rand**	Neutral	643.18 ± 130.654	660.00 ± 113.641	−2.274	0.034*	2.62
	Congruent	783.64 ± 117.333	790.91 ± 119.440	−0.876	0.391	0.93
	Incongruent	871.41 ± 136.070	892.73 ± 136.004	−2.920	0.008**	2.45
**Med**	Neutral	638.64 ± 118.615	617.73 ± 121.653	3.533	0.002**	−3.27
	Congruent	794.55 ± 118.029	764.55 ± 112.238	6.205	<0.001***	−3.78
	Incongruent	865.00 ± 137.797	819.09 ± 133.627	3.302	0.003**	−5.31

*p < 0.05; p < **0.01; ***p < 0.001; repeated measures of ANOVA with Bonferroni adjustment comparing Post values with Pre values. Values are group means ± S.D. Rand—Random Thinking; Med—Meditation.

### Hemodynamic responses in stroop color word task

In the present study, the 16 channel fNIRS device provided a set of time series recorded over the PFC. The locations of the probed regions are shown in Figure 2. The order of the channels is from left to right, i.e., “1” is on the left and “16” is on the right as depicted in Figure 3. Analysis of hemoglobin signals i.e., ΔHbO or ΔHbR is still a controversial issue, specifically which hemoglobin signal is more reliably associated with brain activity still remain unclear (Schroeter et al., 2002). In this study, we have utilized three wavelengths (i.e.,750, 803 and 850 nm). This combination is suitable only for detecting ΔHbO signal. Therefore we used ΔHbO, ΔHbR and ΔTHC signals for statistical analysis. The groups mean values ± S.D. for the ΔHbO, ΔHbR and ΔTHC in stroop task and the two sessions (random thinking and meditation) in “Pre”, “During” and “Post” states are given in Table 3.

**Table 3:** **Group mean values ± S.D. of the oxyhemoglobin (ΔHbO), deoxyhemoglobin (ΔHbR) and total hemoglobin change (ΔTHC) of Stroop color word task before, during and after random thinking (rand) and meditation (Med)**.

Sessions	Voxels	Pre	Stroop_Pre	During				Stroop_Post	Post
				D1	D2	D3	D4
**Oxyhemoglobin (ΔHbO)**									
**Rand**	**Left PFC**	−0.71 ± 3.71	−0.64 ± 7.39	0.51 ± 7.58	0.15 ± 6.69	0.25 ± 7.16	0.21 ± 7.61	0.83 ± 7.41	0.80 ± 7.22
	**Right PFC**	−2.65 ± 5.56	0.81 ± 4.59	−2.21 ± 12.47	−1.30 ± 12.45	−1.69 ± 12.67	−1.65 ± 12.49	−1.56 ± 11.90	−1.00 ± 10.02
**Med**	**Left PFC**	−0.43 ± 6.53	−0.93 ± 2.55	−1.13 ± 3.17	−0.79 ± 3.22	−0.64 ± 3.54	−0.77 ± 3.98	−0.09 ± 5.15	0.44 ± 5.25
	**Right PFC**	−2.45 ± 7.18	−1.30 ± 2.64	−0.71 ± 4.07*	−0.44 ± 3.84*	−0.19 ± 3.86**	−0.89 ± 3.70	−0.79 ± 3.89	0.35 ± 4.41***
**Deoxyhemoglobin (ΔHbR)**									
**Rand**	**Left PFC**	−0.20 ± 15.36	−1.70 ± 4.23	−2.03 ± 5.27	−0.98 ± 5.94	−0.73 ± 6.45	−0.73 ± 6.57	−0.32 ± 8.80	−0.91 ± 8.10
	**Right PFC**	−5.18 ± 10.80	−2.86 ± 3.65	−3.22 ± 6.89	−1.78 ± 5.75***	−0.48 ± 8.08***	0.01 ± 8.05***	1.22 ± 8.18***	0.19 ± 10.25***
**Med**	**Left PFC**	−1.57 ± 6.61	−1.27 ± 8.85	−2.82 ± 18.20	−2.25 ± 18.82	−2.38 ± 19.15	−2.29 ± 18.82	−2.28 ± 19.80	−2.23 ± 17.63
	**Right PFC**	−3.90 ± 8.22	−3.00 ± 7.93	−7.19 ± 23.46	−8.16 ± 23.09	−8.14 ± 23.43	−8.15 ± 22.72*	−7.28 ± 23.56	−7.04 ± 19.93
**Total hemoglobin change (ΔTHC)**									
**Rand**	**Left PFC**	−1.70 ± 5.39	−1.83 ± 9.87	−1.58 ± 20.98	−1.39 ± 21.02	−1.73 ± 21.40	−1.66 ± 21.16	−1.71 ± 21.56	−1.02 ± 19.70
	**Right PFC**	−4.29 ± 6.67	−3.28 ± 9.05	−8.85 ± 28.49	−9.07 ± 27.55*	−10.41 ± 26.99***	−10.28 ± 26.52***	−10.26 ± 26.89**	−8.41 ± 21.55**
**Med**	**Left PFC**	−0.78 ± 17.63	−2.98 ± 7.98	−3.50 ± 9.7	−2.18 ± 10.23	−1.82 ± 10.74	−1.98 ± 11.34	−1.21 ± 14.27	−1.15 ± 13.88
	**Right PFC**	−5.11 ± 11.97	−4.36 ± 5.29	−4.37 ± 7.48	−2.83 ± 7.18**	−1.94 ± 8.48***	−2.16 ± 9.14**	−1.45 ± 10.11**	−0.57 ± 11.07***

**p < 0.01; repeated measures of ANOVA with Bonferroni adjustment comparing During and Post values with Pre values. Values are group means ± S.D.

For ΔHbO, the repeated—measures ANOVA for Sessions (Random thinking and Meditation) × PFC (Left and Right) × States (“Stroop_Pre”, “Stroop_Post”) revealed no significant main effect for Sessions, States and PFC. There was a significant interaction between PFC × States (*F*_(1,175)_ = 9.87, *p* < 0.01, *η*^2^*p* = 0.053); Sessions × PFC × States (*F*_(1,175)_ = 3.17, *p* < 0.01, *η*^2^*p* = 0.040).

For ΔHbR, the repeated—measures ANOVA demonstrated significant main effect for Sessions (*F*_(1,175)_ = 9.99, *p* < 0.01, *η*^2^*p* = 0.054); PFC (*F*_(1,175)_ = 4.57, *p* < 0.05, *η*^2^*p* = 0.025). Also, there was a significant interaction between Sessions × PFC (*F* = 5.11, *p* < 0.05, *η*^2^*p* = 0.028); Sessions × States (*F*_(1,175)_ = 22.13, *p* < 0.001, *η*^2^*p* = 0.112); Sessions × PFC × States (*F*_(1,175)_ = 9.81, *p* < 0.01, *η*^2^*p* = 0.053).

For total hemoglobin (ΔTHC), the repeated—measures ANOVA revealed that there was a significant main effect for PFC (*F*_(1,175)_ = 9.71, *p* < 0.01, *η*^2^*p* = 0.053), and the significant interaction between Sessions × PFC (*F*_(1,175)_ = 5.33, *p* < 0.01, *η*^2^*p* = 0.03); Sessions × States (*F*_(1,175)_ = 19.87, *p* < 0.001, *η*^2^*p* = 0.102); PFC × States (*F*_(1,175)_ = 5.96, *p* < 0.05, *η*^2^*p* = 0.033); Sessions × PFC × States (*F*_(1,175)_ = 14.20, *p* < 0.001, 0.075).

The *post hoc* analysis with Bonferroni corrections demonstrated forehead hemodynamic responses during stroop task related to random thinking and meditation sessions are given in Table 3. The results demonstrated a significant decrease in the concentration of ΔHbO in left PFC (*p* = 0.016) and in the right PFC (*p* = 0.032) after random thinking session during stroop color word task, whereas, there was a significant improvement in ΔHbO in left PFC (*p* = 0.006) and right PFC (*p* = 0.046) following the meditation session.

From the above observations, it can be concluded that meditation enhances bilaterally activation of the anterior PFC and consequently, a stronger increase of oxygenation and cerebral blood flow during stroop task at the right PFC due to interference reduction.

### Hemodynamics responses in meditation and random thinking

For ΔHbO, the repeated—measures ANOVA for Sessions (Random thinking and Meditation) × PFC (Left and Right) × States (Pre Stroop_Pre, D1-D4, Stroop_Post, Post) demonstrated a significant main effects for States (*F*_(7,1225)_ = 5.23, *p* < 0.001, *η*^2^*p* = 0.029). There was a significant interaction between the PFC × States (*F*_(7,1225)_ = 2.42, *p* < 0.001, *η*^2^*p* = 0.014); Sessions × Hemispheres × States (*F*_(7,1225)_ = 7.32, *p* < 0.05, *η*^2^*p* = 0.040).

For ΔHbO, the repeated—measures ANOVA showed there was a significant main effect for Sessions (*F*_(1,175)_ = 12.20, *p* < 0.001, *η*^2^*p* = 0.065); PFC (*F*_(1,175)_ = 7.89, *p* < 0.01, *η*^2^*p* = 0.043) and States (*F*_(7,1225)_ = 3.55, *p* < 0.001, *η*^2^*p* = 0.019). There was a significant interaction between the Sessions × PFC (*F*_(1,175)_ = 4.13, *p* < 0.001, *η*^2^*p* = 0.023); Sessions × States (*F*_(7,1225)_ = 9.99, *p* < 0.001, *η*^2^*p* = 0.054); Sessions × PFC × States (*F*_(7,1225)_ = 10.37, *p* < 0.001, *η*^2^*p* = 0.056).

For total hemoglobin change (ΔTHC), there was a significant main effect for Sessions (*F*_(1,175)_ = 5.07, *p* < 0.05, *η*^2^*p* = 0.028); PFC (*F*_(1,175)_ = 12.20, *p* < 0.001, *η*^2^*p* = 0.065); and States (*F*_(1,175)_ = 2.79, *p* < 0.01, *η*^2^*p* = 0.016) and a significant interaction between the Sessions × PFC (*F*_(1,175)_ = 6.45, *p* < 0.05, *η*^2^*p* = 0.036); Sessions × States (*F*_(7,1225)_ = 9.06, *p* < 0.001, *η*^2^*p* = 0.049); PFC × States (*F*_(7,1225)_ = 2.34, *p* < 0.05, *η*^2^*p* = 0.036]; Session × PFC × State (*F*_(7,1225)_ = 14.51, *p* < 0.001).

*Post hoc* analyses with Bonferroni corrections were performed on ΔHbO, ΔHbR and ΔTHC and all comparisons were made with respective “Pre” state. These have been summarized in Table 3. There was a significant increase in ΔHbR at the right PFC (*p* = 0.005) after random thinking session whereas there was a significant increase in the left PFC (*p* = 0.02) and in right PFC (*p* < 0.001) after meditation session. Similarly, in ΔTHC, there was a significant decrease in blood flow change in the right PFC (*p* < 0.001) after the random thinking session whereas there was a significant increase in blood flow change in the left (*p* = 0.03) and in right PFC (*p* < 0.001) after meditation session.

**Figure 5:**
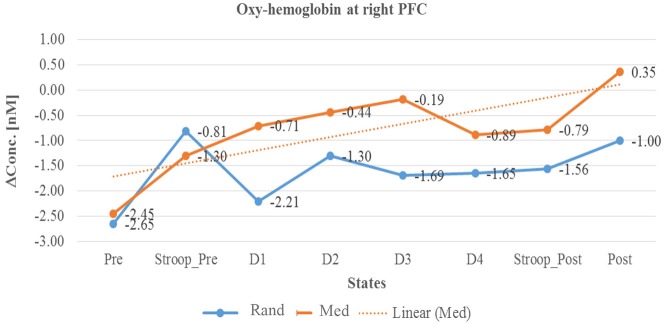
**Line graph represents averaged Oxy-hemoglobin change at right prefrontal cortex (rPFC) in two sessions i.e., random thinking and meditation and Stroop task. Note**: Line graph represents comparisons between baseline, stroop_pre, during sessions (random thinking and meditation), stroop_post, and post. Stroop Pre showed higher Oxy-hemoglobin change compared to baseline. During and after meditation, the cerebral oxygenation was higher in rPFC compared to random thinking.

**Figure 6:**
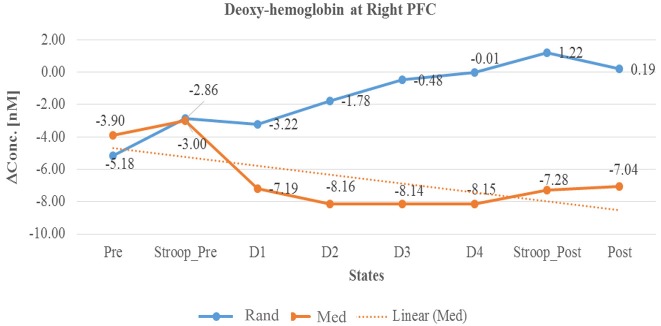
**Line graph represents averaged Deoxy-hemoglobin change at right PFC in two sessions i.e., random thinking and meditation and Stroop task. Note**: Line graph represents de-oxyhemoglobin changes was higher in right PFC during random thinking (D2, D3, and D4), stroop task and after random thinking. In other hand, during meditation, there was a decrease in de-oxyhemoglobin in D3 level in rPFC.

**Figure 7:**
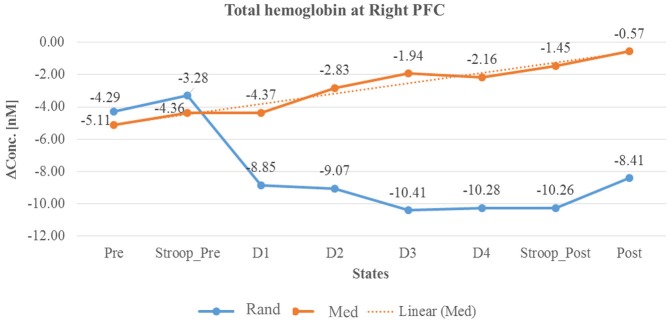
**Line graph represents averaged total hemoglobin change at rPFC in two sessions i.e., random thinking and meditation and Stroop task. Note**: Line graph represents total hemoglobin change was higher in rPFC during meditation (D2, D3, and D4), in stroop task, and in post session. In other hand, there was a decrease in rPFC during random thinking (D2, D3, and D4), in stroop task and in post session.

In summary, as described in Table 3 and in Line diagrams (Figures 5–7), there was a positive trend to show a significant increase in the concentration of oxyhemoglobin change (ΔHbO) during meditation session at right PFC (as shown in Figure 5). There was a significant decrease in deoxyhemoglobin change (ΔHbR) (as shown in Figure 6) during meditation session whereas there was a significant increase in the concentration of deoxyhemoglobin change during random thinking session at the right PFC. Additionally, there was also a significant increase in the total hemoglobin change (ΔTHC) during and after meditation sessions (Figure 7) and decrease in the total hemoglobin change (ΔTHC) during and after random thinking session.

## Discussion

The primary goal of the present study was to ascertain whether meditation increases rCBF at bilateral PFC, measured with fNIRS, compared to random thinking. Our secondary goal was to observe the RT scores and relative changes in cerebral blood flow, and to determine if there are persistent effects following meditation session compared to random thinking session. Results as confirmed with recent studies on meditation with spectroscopy (Cheng et al., 2010), SPECT imaging (Newberg et al., 2001, 2010a,b; Cohen et al., 2009) and fMRI (Short et al., 2010; Guleria et al., 2013; Zeidan et al., 2014) have revealed that meditation program resulted in significant increases in baseline CBF ratios in the prefrontal, superior, inferior and orbital frontal cortex, dorsolateral prefrontal cortex (DLPFC), right dorsal medial frontal lobe, cingulate gyrus and right sensorimotor cortex. In present study, we found that brain activation, measured by changes in ΔHbO and ΔTHC concentration in the right prefrontal area was followed by a strong decrease in ΔHbR concentration during meditation. Additionally, the rCBF significantly increased in the right frontal lobe during stroop task after meditation, which suggest the improvement in the participant’s performance (reaction time) during the task. The total blood oxygenation (ΔTHC) level in the PFC could rise with increasing task load from neutral to congruent, and then incongruent; this would demonstrate a positive correlation with performance measures. The changes in regional blood flow is mediated by changes in neural activity in a single region or in several selective regions of the brain (Lauritzen, 2001).

Earlier studies have demonstrated that the PFC is activated particularly on the right PFC and anterior cingulate cortex (ACC) in willful act and tasks that require intense focused and sustained attention (Frith et al., 1991; Pardo et al., 1991; Vogt et al., 1992; Petersen and Posner, 2012). A study on eight Tibetan Buddhist meditators demonstrated improved activity in the PFC bilaterally (though greater on the right hemisphere) and the cingulate gyrus during meditation (Newberg and Iversen, 2003). This suggests that meditation begins with activation of the PFC and anterior cingulate gyrus associated with the will or intent to clear the mind of thoughts or to focus on an object (Edwards et al., 2012).

Meditation increases CBF and decreases cerebrovascular resistance (CVR) suggesting a contributing vascular mechanism (Jevning et al., 1996) which reflect cerebral activation. The CVR reduction being associated with cognitive improvement which suggests a vascular contribution to cognitive enhancement (Nation et al., 2013). During meditation, the activation of right PFC is theoretically associated with the activity in the reticular nucleus of the thalamus. This activation may be accomplished by the PFC’s production and distribution of glutamate, a known excitatory neurotransmission (Cheramy et al., 1987; Finkbeiner, 1987), which communicate with other brain structures such as lateral geniculate and lateral posterior nuclei of the thalamus (Portas et al., 1998). An early study on meditation with single photon emission computed tomography (SPECT) demonstrated a general increase in thalamic activity that was proportional to the activity levels in the PFC (Newberg et al., 2001; Edwards et al., 2012). The activation on the right PFC causes increased activity in the reticular nucleus during meditation, the results may be decreased sensory input entering into the posterior superior parietal lobule which is involved in the analysis and integration of higher order visual, auditory, and somesthetic information (Adair et al., 1995).

A major strength of the present study was to examine the states of meditation and random thinking related hemodynamic responses in cerebral oxygenation during performance of the stroop color word task. It is a well established phenomenon that executive processes are facilitated by the frontal lobe and due to stroop interference brain activity may depend on increased ability to recruit frontal neural resources (Schroeter et al., 2004b). This allowed us to examine whether there is an increase in oxygenation with meditation corresponding to an ability to recruit appropriate resources for task performance or a decrease in activation corresponding to better optimization and possible reduction in task difficulty with meditation. In a study, fNIRS showed stroop interference is consistently associated with the ACC and the lateral prefrontal cortex (LPFC), especially the DLPFC, where the ACC is considered to be susceptible to conflict, and the DLPFC is purported to implement cognitive control (Carter et al., 2000; Leung et al., 2000). DLPFC may involve attentional maintenance while ACC monitors performance (MacDonald et al., 2000). Another similar study suggested meditation may enhance specific subcomponents of attention such as conflict monitoring or performance (Jha et al., 2007). Although fNIRS cannot monitor the cortical activation in the ACC because its measurement is limited to lateral cortical surfaces, it has successfully monitored the activation of the LPFC associated with stroop interference (Schroeter et al., 2002, 2003, 2004a,b; Ehlis et al., 2005).

There have been several neuroimaging studies evaluating the cerebral blood flow and performance of different meditation practices using behavioral, EEG and (Carter et al., 2005) fMRI imaging. Previous studies on meditation and EEG reported, greater midline theta power and slow alpha power in the frontal area during meditation (Takahashi et al., 2005; Chan et al., 2008). Zazen meditation showed increased alpha-1 and alpha-2 frequency activity of EEG in right prefrontal areas including insula, parts of the somatosensory, motor cortices and temporal areas (Faber et al., 2014). A subsequent study, on Satyananda Yoga meditation practice, showed greater source activity in low frequencies (particularly theta and alpha 1) during mental calculation, body-steadiness and mantra meditation (Thomas et al., 2014). Additionally, body-steadiness and mantra meditation showed greatest activity in right side of superior frontal and precentral gyri, parietal and occipital lobes. Similarly, neuroimaging studies on meditation practice, when compared to the control session showed significantly increased oxy-hemoglobin and CBF in the medial PFC which was associated with the intense focus-based component of the practice (Wang et al., 2011). Meditation involves attentional regulation and leads to increased activity in brain regions associated with attention such as DLPFC and ACC. The long-term practitioners had significantly more consistent and sustained activation in the DLPFC and the ACC during meditation vs. control in comparison to short-term practitioners (Baron Short et al., 2010). These studies suggest that willful acts and tasks that require sustained attention are initiated via activity in the PFC, particularly in the right hemisphere (Posner and Petersen, 1990; Frith et al., 1991; Pardo et al., 1991; Ingvar, 1994). Meditation requires focus of attention on objects which thereby activates PFC, particularly in the right hemisphere (Cohen et al., 2009), as well as the cingulate gyrus (Herzog et al., 1990; Lazar et al., 2000; Newberg et al., 2001). This demonstrated that during meditation there was an increased activity in the PFC bilaterally (greater on the right) and the cingulate gyrus (Newberg and Iversen, 2003). Therefore, the process of meditation seems to happen by activation of the prefrontal and cingulate cortex which are associated with the will or intent to clear one’s mind of thoughts or to focus on an object.

In other imaging studies on meditation, there have been inconsistent results regarding the frontal cortex. A recent study showed decreased frontal activity during externally guided word generation compared to internal or volitional word generation (Cross et al., 2012). Thus, prefrontal and cingulate activation may be associated with the volitional aspects of meditation. Meditation with fluorodeoxyglucose (FDG) PET in eight subjects undergoing Yoga meditative relaxation (Herzog et al., 1990) reported increased rCBF in the frontal: occipital ratio of cerebral metabolism. Specifically, there was a mild increase in the frontal lobe, but marked decreases in metabolism in the occipital and superior parietal lobes. In addition to these studies, the PFC is reported to have a crucial role in social cognitive skills and along with the cingulate gyrus governs social behavior tasks related to Theory of Mind, empathy, moral reasoning, and evaluation of emotional states (Declerck et al., 2006). The PFC is essential for flexible behavior because it inhibits the habitual responses that have become inappropriate (Mesulam, 1998). But, an increase in the activity of PFC (determined by fNIRS) is not necessarily beneficial always. For example, animal experimentation has shown that the electrical activation of the medial PFC prevent the proper sequence of pressing the lever and collecting the reward (a pellet of food) in an operant condition task (Cross et al., 2012; Jurado-Parras et al., 2012) and also prevent the expression of an already acquired classically conditioned eyelid response (Leal-Campanario et al., 2007, 2013). However, in our study we infer that activation of prefrontal cortices after meditation had beneficial effects on cognition as manifested by improved performance in stroop color word task.

The present study reported increased oxy-hemoglobin concentration because of enhanced neural activity and cerebral blood flow in the prefrontal area during meditation compared to random thinking. In such studies, it is very important to understand the influences of systemic artifacts such as those from the heart, breathing, superficial perfusion, etc., which may be induced by the cognitive tasks related stress and autonomic responses. For example, a recent study performed on peripheral physiological measurements with temporal correlations of fNIRS and fMRI signals concluded that the physiological basis of the systemic artifact is a task-evoked sympathetic arterial vasoconstriction monitored by a decrease in venous volume and these artifacts are fairly common (Kirilina et al., 2012). They also suggested that the separation of fNIRS signals originating from activated brain and from scalp is a necessary precondition for unbiased fNIRS brain activation maps and pre-processing of the raw data using high definition filters is necessary.

In summary, the results of the present study provided first evidence that the oxygenation levels are increased in the PFC during meditation compared with random thinking in the same practitioners. Further event-related NIRS studies may apply well-tested fMRI paradigms in studies with children and patients, utilizing the advantages of the method.

## Conflict of interest statement

The authors declare that the research was conducted in the absence of any commercial or financial relationships that could be construed as a potential conflict of interest.

## Footnotes

^1^http://www.randomizer.org
